# Nutritional anthropometry of children and adolescents in Spain in the middle of the 20th century (1934–1965)

**DOI:** 10.1017/S1368980026102250

**Published:** 2026-03-31

**Authors:** María Dolores Marrodán, Ana Garcés Sánchez, María Sánchez Álvarez, Angel Herráez, Marisa González Montero de Espinosa

**Affiliations:** 1 Department of Biodiversity, Ecology and Evolution, Complutense University of Madrid, Spain; 2 Research Group for Nutritional Epidemiology, Complutense University of Madrid, Spain; 3 Faculty of Health Sciences, Faculty of Humanities and Social Sciences, Isabel I of Castile International University, Spain; 4 Department of Systems Biology, University of Alcalá, Spain

**Keywords:** Malnutrition, Underweight, Weight, Height, BMI, Secular trend, Stunting

## Abstract

**Objective:**

The general objective was to explore the nutritional condition of schoolchildren, based on certain body measurements published in various reports (from 1934 to 1965). More specifically, we intended to analyse, first, the secular trend of growth by comparing historical anthropometric data with current national and international figures and, second, to study the possible variability of body measurements at that time among different Spanish regions and of different socioeconomical levels.

**Design:**

Based on these reports, a sample of individuals of both sexes, between 6 and 16 years of age, was selected. Average weight and height were calculated for each age and sex, and the BMI was determined for all series. The data of these series were compared first with each other and then, using the z-score method, with reference tables published by the WHO and by a current Spanish reference.

**Setting:**

Seven Spanish historical series, from 1934 to 1965.

**Subjects:**

114 880 individuals aged 6 to 16 years (59 786 boys and 55 094 girls).

**Results:**

Almost all the historical populations assessed show chronic undernutrition and underweight, strongly influenced by socioeconomic status. On the other hand, nutritional status appears almost independent of rural or urban environment.

**Conclusions:**

In the series analysed, various states of chronic undernutrition and underweight were present, mainly in schoolchildren from the most disadvantaged social groups. The secular trend in height and weight occurred in times after the beginning of Franco’s developmentalism.

The Spanish economy in the 20th century can be divided into several distinct phases: during the first third of the century, growth was similar to that of other European countries; stagnation set in during the 1930s and 1940s, lasting well into the 1950s; there was a major upheaval throughout the 1960s, with rapid development that, despite some ups and downs, continued and even intensified in the last quarter of the century^(1)^. At the beginning of the 1930s, the Spanish economy faced several problems: some of them came from Primo de Rivera’s dictatorship (1923–1930) and others were caused by the Second Republic (1931–1936). Both were aggravated by the late impact of the October 1929 crash, produced by the fall of the New York Stock Exchange. This crisis, together with social and political problems, led to the Spanish Civil War (1936–1939)^(2)^.

The general who emerged victorious from that conflict, Francisco Franco, established a dictatorial regime that lasted several decades, until his death in 1975. The post-war period was characterised by political, social and economic isolation, which hindered recovery of the country. It was a time when Franco’s regime was internationally isolated, supported economically only by the Argentinian government of Juan Domingo Perón, at the end of the 1940s^(3)^. The significant food shortages gave rise to what is now known as the ‘Spanish famine’^(4,5)^. This is evident in the cooking habits in the post-war period, with a great scarcity of materials and an abundance of ingenuity and imagination^(6)^. Living conditions for most Spaniards, more so for the working classes, were very poor and with a practically chronic caloric deficit^(7)^.

The shortage of housing, the sanitary hygienic deficit and the insufficient quantity and diversity of food contributed to increased morbimortality and prevalence of infectious and deficiency diseases. Certain neurological disorders related to malnutrition^(8)^ were triggered; these included the pellagra epidemic (winter of 1937–1938), caused by a shortage of vitamin B3^(9)^, and the so-called Vallecas syndrome (1941–1942), which produced cramps and muscle weakness due to a lack of B complex vitamins^(10)^. Finally, the chronic intoxication called lathyrism^(11)^ should also be mentioned, which was generated by an excessive consumption of flour from grass pea (*Lathyrus sativus*), rich in a neurotoxic amino acid.

Nutritional studies carried out during the post-war period^(12,13)^ showed that the social classes with lower purchasing power did not meet the energy, protein, mineral and vitamin requirements (especially calcium and vitamins A and B2). Social inequalities were very noticeable, which translated into important differences in the coverage of nutritional needs, such that 45 % of families with low purchasing power did not reach 50 % of the minimum caloric needs, while only 10 % of those with the highest socioeconomic level did not reach this threshold^(14)^.

As a consequence of the food shortage in 1939, ration cards were implemented throughout the country, with the objective of ensuring the basic necessities in the population. These cards, which were in force until 1953, allowed some families to survive, although in many cases they did not cover their food needs, and, therefore, it was common to resort to the practice of ‘estraperlo’ (black market)^(15)^.

Beginning in 1959, a series of economic reforms (known as the Stabilisation Plan) were carried out which, over the following years, produced a great change in the country’s productive structure. This led to the so-called ‘Spanish economic miracle’, which took place from the beginning of the 1960s onwards. This recovery is mainly attributed to the technological update, the inflow of foreign currency from tourism and the return of Spanish emigrants, as well as foreign credits and investments^(16)^. All this led to an increase in daily caloric intake per inhabitant in the sixth decade of the 20th century compared to previous years^(17)^. Thus, the 1965 Family Budget Survey showed that the nutritional situation of the Spanish population was adequate for most nutrients, except for vitamins A and B2^(18)^. All this political and economic development had a direct impact on the nutritional status of the Spanish population.

Anthropometry is a valuable tool to reflect the degree of nutritional adequacy of individuals. It is possible to establish the level of child and adolescent growth through body size and composition. Thus, quantification of certain variables, such as height and weight, allows, on the one hand, to diagnose pathological cases of malnutrition or underweight and, on the other hand, to determine the secular trend of the aforementioned anthropometric magnitudes. It may be noted that there exist historical anthropometric data in Spain dating back to the 19th century, arising from the summer school camps organised by *Institución Libre de Enseñanza* (the Free Institution of Education) and which have been previously analysed^(19–22)^.

## Objectives

The general objective of the present work was to analyse the nutritional condition of Spanish schoolchildren from the times of the Republic to the Francoist developmentalism, based on available historical anthropometrical reports. In addition, two other specific objectives were also proposed: first, to determine the secular trend of growth by comparing the anthropometric data in the period 1944–1965 with the corresponding pre-war values (1934); second, to contrast the ancient values with the current ones, both nationally and internationally, and third, to study the possible variability of body measurements at the aforementioned period among different Spanish regions and at different socioeconomical levels.

## Material and methods

The sample for this study proceeds from the only seven published reports by Spanish authors found among the literature of the period, which report anthropometric measurements of children and adolescents from different regions and different socioeconomic levels and environments (Table 1). One comes from the Spanish Second Republic period, a study published in 1934 (without the author specifying the year when data were actually collected) and carried out with schoolchildren from peri-urban areas in León and of middle socioeconomic status^(23)^. Five others focus on schoolchildren in the post-war period, published between 1944 and 1953. Those from Madrid^(24)^ and Barcelona^(25)^ were composed of two series with respective low and high socioeconomic status. In the first case, schoolchildren from Vallecas, a socially deprived neighbourhood on the outskirts of the capital, were compared to others attending private schools located in Chamberí, a middle-class quarter. Finally, three other studies were conducted by the same researchers with data collected at different locations in Spain, one in a rural area in Asturias (northern Spain)^(26)^ and two in southern Spain: Málaga^(27)^ and Granada^(28)^. Finally, a report elaborated in the 1960s^(29)^ corresponds to the first study in Spain that analyses the height and weight of 128 317 schoolchildren of both sexes from all over the country.

**Table 1.** Reports included in the current analysis, published between 1934 and 1965

Ref.	N	Date of data collection	Population	Environment, socioeconomic level	Sex	Age (years old)
**1.**	540	Unknown	León	Suburban, medium	m & f	6–14
**2.a**	210	1941–1942	Puente de Vallecas (Madrid)	Urban, low	m	5–13
**2.b**	67	1941–1942	Chamberí (Madrid)	Urban, medium-high	m	5–16
**3.**	1530	1942–1946	Mieres (Asturias)	Rural, medium	m & f	1–22
**4.a**	726	1944–1945	Barcelona	Urban, low	m & f	7–14
**4.b**	751	1944–1945	Barcelona	Urban, medium-high	m	7–14
**5.**	1040	1942–1946	Málaga	Urban, medium-low	m	11–19
**6.**	2015	1943–1946	Granada	Urban, medium-low	m	10–20
**7.**	128 317	1963–1964	whole Spain	Rural, low	m & f	4–14

**1.** Morros Sardá, 1934^(23)^; the author does not refer the year when data were collected. **2.** Grande Covián et al., 1944^(24)^. **3.** Fernández Cabezas & Fernández Cabezas, 1946^(26)^**. 4.** Prevosti, 1949^(25)^. **5.** Fernández Cabezas & Fernández Cabezas, 1952^(27)^. **6.** Fernández Cabezas & Fernández Cabezas, 1953^(28)^. **7.** Palacios & Vivanco (1965) ^(29)^. All these studies were transversal.

In some cases, these studies collected data from more than one school group, and therefore in Table 1 the reports have been identified with reference number and in chronological order. From these studies, a sample of individuals of both sexes, aged 6 to 16 years only, was extracted. The mean values of weight and height, as well as their sd, were collected for each age and sex. From these data that made up the sample, the average BMI was calculated for all the series analysed.

In order to establish nutritional status, the mean anthropometric values (height for the age, weight for the age) of the aforementioned historical series were compared with two current sources. The first one was the international growth reference of the WHO^(30)^, collected in the 1970s, the preparation of which is described by de Onis *et al.* ^(31)^. Only height and BMI could be used in this case, since this institution reports weight data only below 10 years of age. The second comparison was against recent Spanish data obtained from middle-class schoolchildren residing in the city of Madrid and of Spanish ancestry^(32)^. Divergence between each historical series and the more recent reference values, either national or international, was assessed by the value of z-score, which indicates the multiple of sd a data diverge from the mean value of the reference population. In the case of comparison to WHO reference, z-scores were calculated using the AnthroPlus software^(33)^. Z-score values are then presented here using a gradient of colour intensities in heat map plots.

The z-scores were then classified according to the established criteria^(34)^: Height for age: z between –1 and –2, mild chronic malnutrition; z between –2 and –3, moderate chronic malnutrition; z below –3, severe chronic malnutrition; weight for age: z between –1 and –2, mild underweight; z between –2 and –3, moderate underweight; z below –3, severe underweight; BMI for age: z between –1 and –2, mild acute malnutrition; z between –2 and –3, moderate acute malnutrition; z below –3, severe acute malnutrition.

Statistical analyses were performed using SPSS (IBM Statistical Package for Social Sciences, version 26).

## Results

### Height for age

With respect to height in the male sex, Table 2 allows to appreciate that participants living in suburban and rural areas (León and Mieres) had the lowest values, together with those from the Vallecas quarter in Madrid. We then analysed height among male children and youngsters in Barcelona^(25)^, finding differences between the two samples of different socioeconomic level throughout the entire period covered by the studies.

**Table 2.** Height of Spanish schoolchildren (cm), male series. Mean and sd, when available, extracted from each published article (references described in Table 1)

		Years of age										
Ref.		6	7	8	9	10	11	12	13	14	15	16
**1.**	m	109·7	117·6	121·2	123·4	130·6	132·7	138·0	139·7	144·5	–	–
	sd	7·9	4·9	5·6	6·3	6·2	6·4	7·6	7·2	7·1	–	–
**2.a**	m	107·8	110·3	113·9	118·6	119·8	126·2	129·6	129·8	–	–	–
**2.b**	m	116·3	119·7	126·3	134·3	136	140·8	150·2	147·1	162·6	158·4	161·4
**3.**	m	106·6	112·7	116·0	121·2	126·6	129·8	133·6	139·2	145·0	152·1	159·0
	sd	9·3	10·1	8·16	6·2	7·9	7·0	8·3	9·6	9·3	10·4	9·1
**4.a**	m	–	116·4	123·3	125·0	130·1	133·2	137·9	142·6	147·2	–	–
	sd	–	5·8	5·5	5·3	6·0	6·3	5·9	8·3	8·7	–	–
**4.b**	m	–	121·2	127·0	131·4	137·2	140·9	145·9	150·8	156·2	–	–
	sd	–	4·9	5·1	5·6	5·8	5·8	6·5	8·4	8·5	–	–
**5.**	m	–	–	–	–	–	131·0	135·9	140·3	148·8	156·0	159·4
**6.**	m	–	–	–	–	128·8	132·2	135·7	139·8	143·8	152·2	158·0
	sd	–	–	–	–	6·9	5·1	9·2	10·3	9·0	10·4	10·4
**7.**	m	113·2	118·3	124·4	128·3	132·7	137·2	141·8	146·9	152·8	–	–

**1.** Morros Sardá, 1934^(23)^, suburban, middle socioeconomic status (SES). **2.** Grande Covián et al., 1944^(24)^; (a) urban, low SES; (b) urban, mid-high SES. **3.** Fernández Cabezas & Fernández Cabezas, 1946^(26)^, rural, middle SES. **4.** Prevosti, 1949^(25)^; (a) urban, low SES; (b) urban, mid-high SES. **5.** Fernández Cabezas & Fernández Cabezas, 1952^(27)^, urban, mid-low SES. **6.** Fernández Cabezas & Fernández Cabezas, 1953^(28)^, urban, mid-low SES. **7.** Palacios & Vivanco (1965)^(29)^, rural, low SES.

It is important to note that male individuals in the most recent report^(29)^, covering locations all over Spain, were taller than those in all the previous series at any age, with the exception of the medium-high social status samples in Madrid and Barcelona. This is easily explained by the fact that the publication corresponds to the 1960s, i.e. the beginning of Franco’s developmentalism.

With regard to females (Table 3), despite having less information available, the same trend is observed: the average height of girls was lower in rural and suburban environments than in urban areas. The exception here, as in the case of boys, is that female height in rural areas throughout Spain, the series from Palacios et al.^(29)^ included in the comparison, was higher than in urban areas (except in the upper-middle strata of Madrid and Barcelona). This is due to two factors: the first already mentioned above with respect to the time of publication and second that certain regions in our country (Levante, Catalonia, Cantabria, Balearic Islands) had a higher economic development, a fact that influences the average height.

**Table 3.** Height of Spanish schoolchildren (cm), female series. Mean and sd, when available, extracted from each published article (references described in Table 1)

		Years of age										
Ref.		6	7	8	9	10	11	12	13	14	15	16
**1.**	m	108·7	115·8	121·0	124·6	132·1	134·2	139·8	143·7	149·8	–	–
	sd	4·4	5·0	5·9	5·2	6·1	5·2	7·7	6·5	6·3	–	–
**3.**	m	107·7	112·3	116·9	121·7	125·6	129·6	134·3	141·7	146·0	153·3	154·9
	sd	6·1	7·9	3·6	7·8	7·1	7·2	8·1	7·9	9·1	6·6	6·7
**4.a**	M	–	117·1	122·2	125·3	130·6	135·6	140·8	146·7	149·7	–	–
	sd	–	5·4	5·5	6·3	7·3	7·1	7·2	6·9	6·4	–	–
**7.**	m	112·1	117·0	122·0	127·5	132·3	137·3	142·7	146·8	151·1	–	–

**1.** Morros Sardá, 1934^(23)^, suburban, middle socioeconomic status (SES). **3.** Fernández Cabezas & Fernández Cabezas, 1946^(26)^, rural, middle SES. **4.a** Prevosti, 1949^(25)^, urban, low SES. **7.** Palacios & Vivanco (1965)^(29)^, rural, low SES.

**Figure 1.**
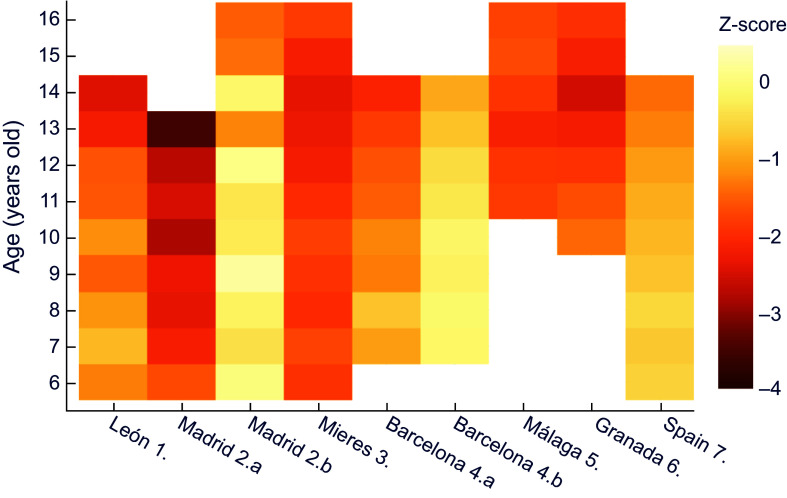
Z-score values for males’ height for age, relative to WHO^(30)^. **1.** Morros Sardá, 1934^(23)^, suburban, middle socioeconomic status (SES). **2.** Grande Covián et al., 1944^(24)^; (a) urban, low SES; (b) urban, mid-high SES. **3.** Fernández Cabezas & Fernández Cabezas, 1946^(26)^, rural, middle SES. **4.** Prevosti, 1949^(25)^; (a) urban, low SES; (b) urban, mid-high SES. **5.** Fernández Cabezas & Fernández Cabezas, 1952^(27)^, urban, mid-low SES. **6.** Fernández Cabezas & Fernández Cabezas, 1953^(28)^, urban, mid-low SES. **7.** Palacios & Vivanco (1965)^(29)^, rural, low SES.

Next, comparisons were made for each historical population against a common, more recent, reference dataset, trying to establish nutritional status. A comparison of z-scores for male height for age in the historical series against data provided by the WHO^(30)^ shows that the lowest z values correspond to Vallecas quarter in Madrid, with an extreme of –3·5 at 13 years of age (Figure 1). This area, located on the outskirts of the capital, was one of the most economically depressed at the time. It should also be noted that the values obtained exceed the threshold indicating moderate chronic malnutrition (z between –2 and –3) at all ages except for six years old.

On the other hand, Figure 1 also shows that boys from Mieres, León, Málaga, Granada and the socially deprived series from Barcelona (which the author called ‘humble’) showed diagnostic values of at least mild retarded growth (z between −1 and −2), with the exception of children from Barcelona and León aged 7 and 8 years, respectively. Data for Spain as a whole^(29)^ and for the upper-middle class in Madrid^(24)^ showed mild stunting at older ages. In contrast, the higher class (‘affluent’) Barcelona series did not show any values of z below −1.

This way of comparing against a common reference also allows to clearly perceive the influence of socioeconomic status (that was noted in Table 2) in the same location and period, namely the two subpopulations in each of the reports from Madrid^(24)^ and Barcelona^(25)^.

**Figure 2.**
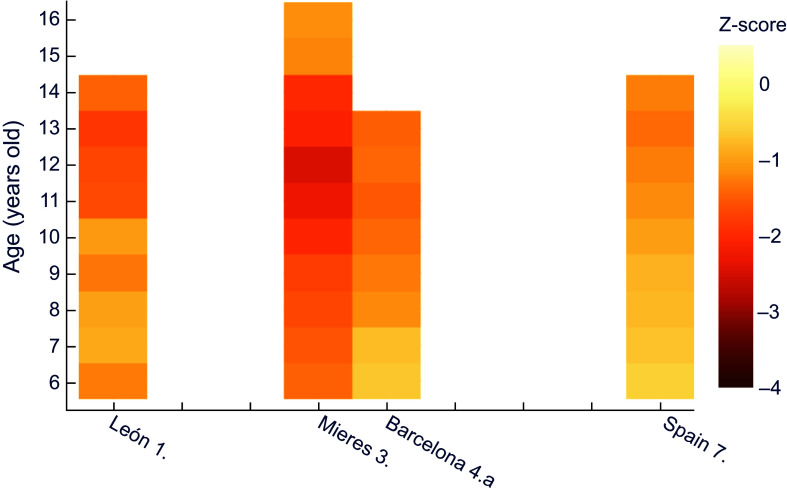
Z-score values for females’ height for age, relative to WHO^(30)^. **1.** Morros Sardá, 1934^(23)^, suburban, middle socioeconomic status (SES). **3.** Fernández Cabezas & Fernández Cabezas, 1946^(26)^, rural, middle SES. **4.a** Prevosti, 1949^(25)^, urban, low SES. **7.** Palacios & Vivanco (1965)^(29)^, rural, low SES.

Similarly, for female height it can be observed (Figure 2) that girls of all ages in Mieres obtained values of z-score below –1 with respect to the international reference; this would correspond to stunted growth, which evolved into moderate chronic malnutrition between 10 and 13 years of age. Over practically the whole age range, the population of León shows mild chronic malnutrition. The same occurs in schoolchildren of low socioeconomic status from Barcelona^(25)^ and in Spain as a whole^(29)^, from 8 and 10 years of age, respectively.

**Figure 3.**
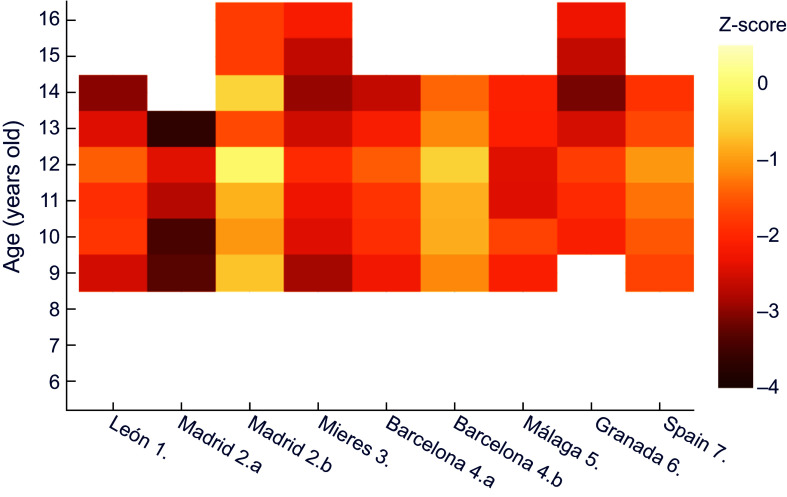
Z-score values for males’ height for age, relative to the national reference^(32)^. **1.** Morros Sardá, 1934^(23)^, suburban, middle socioeconomic status (SES). **2.** Grande Covián et al., 1944^(24)^; (a) urban, low SES; (b) urban, mid-high SES. **3.** Fernández Cabezas & Fernández Cabezas, 1946^(26)^, rural, middle SES. **4.** Prevosti, 1949^(25)^; (a) urban, low SES; (b) urban, mid-high SES. **5.** Fernández Cabezas & Fernández Cabezas, 1952^(27)^, urban, mid-low SES. **6.** Fernández Cabezas & Fernández Cabezas, 1953^(28)^, urban, mid-low SES. **7.** Palacios & Vivanco (1965)^(29)^, rural, low SES.

When comparing against the contemporary Spanish reference^(32)^ (Figure 3), similar results were obtained for boys – even more extreme – than those in comparison with the international reference. Thus, the most disadvantaged sector in Madrid at age 13 exceeded a z-score of –3·5, and all the historical series presented at least mild stunted growth, except for some ages in the economically more favoured series of Barcelona^(25)^ and Madrid^(24)^.

**Figure 4.**
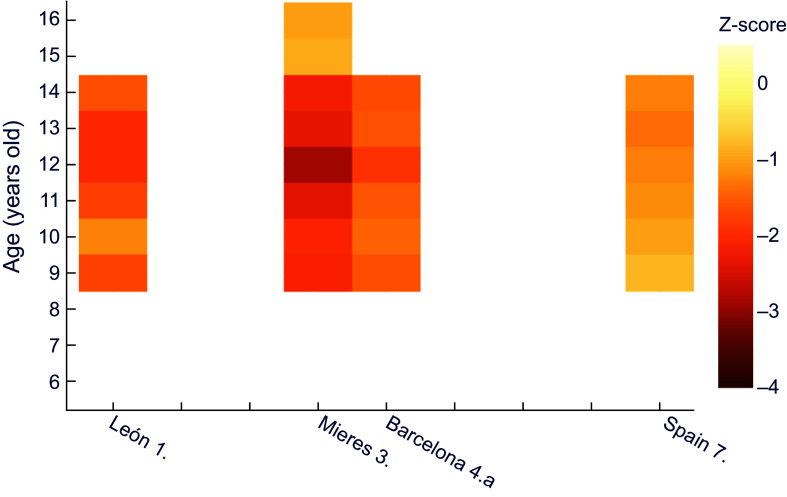
Z-score values for females’ height for age, relative to the national reference^(32)^. **1.** Morros Sardá, 1934^(23)^, suburban, middle socioeconomic status (SES). **3.** Fernández Cabezas & Fernández Cabezas, 1946^(26)^, rural, middle SES. **4.a** Prevosti, 1949^(25)^, urban, low SES. **7.** Palacios & Vivanco (1965)^(29)^, rural, low SES.

For female height for age, z-scores in comparison to the recent Spanish reference (Figure 4) were similar to boys, with all schoolgirls showing mild chronic malnutrition except for the 9-year-old girls in the study covering the whole country^(29)^. Likewise, female individuals from Mieres showed z values under –2 between 6 and 14 years of age.

### Weight for age

Findings for weight are similar to those for height, i.e. both boys and girls (Tables 4 and 5) show lower values in rural and suburban areas than in urban areas, with the same exceptions. There were marked differences in weight between boys of different socioeconomic levels in Barcelona, as was the case for height.

**Table 4.** Weight of Spanish schoolchildren (kg), male series. Mean and sd, when available, extracted from each published article (references described in Table 1)

		Years of age										
Ref.		6	7	8	9	10	11	12	13	14	15	16
**1.**	m	19·2	21·4	23·3	24·8	28·0	29·0	33·9	36·0	41·4	–	–
	sd	2·3	1·8	2·2	2·7	3·4	3·5	6·5	4·8	5·7	–	–
**2.a**	m	17·4	17·6	18·4	20·5	21·0	23·2	25·7	25·1	–	–	–
**2.b**	m	21·6	22·4	24·1	28·8	31·5	35·1	38·5	38·1	50·2	46·9	50·5
**3.**	m	18·6	20·0	22·8	24·9	27·0	29·1	30·8	33·4	38·9	44·5	51·5
	sd	2·6	2·4	3·6	2·6	3·7	3·7	4·1	4·5	6·6	7·1	6·6
**4.a**	m	–	22·3	24·9	25·3	28·2	28·7	32·3	36·1	39·2	–	–
	sd	–	2·9	3·1	3·1	3·4	3·8	3·7	6·4	7·5	–	–
**4.b**	m	–	24·9	28·7	30·9	33·9	36·0	39·2	42·1	49·6	–	–
	sd	–	2·2	3·5	3·7	5·8	5·1	6·2	8·5	9·9	–	–
**5.**	m	–	–	–	–	–	27·1	30·4	32·7	38·2	44·6	47·6
**6.**	m	–	–	–	–	26·6	28·5	30·4	33·4	35·8	41·9	47·6
	sd	–	–	–	–	3·3	3·9	8·6	6·1	6·7	8·7	8·8
**7.**	m	20·7	22·8	25·3	27·8	32·4	32·8	35·4	39·1	41·1	–	–

**1.** Morros Sardá, 1934^(23)^, suburban, middle socioeconomic status (SES). **2.** Grande Covián et al., 1944^(24)^; (a) urban, low SES; (b) urban, mid-high SES. **3.** Fernández Cabezas & Fernández Cabezas, 1946^(26)^, rural, middle SES. **4.** Prevosti, 1949^(25)^; (a) urban, low SES; (b) urban, mid-high SES. **5.** Fernández Cabezas & Fernández Cabezas, 1952^(27)^, urban, mid-low SES. **6.** Fernández Cabezas & Fernández Cabezas, 1953^(28)^, urban, mid-low SES. **7.** Palacios & Vivanco (1965)^(29)^, rural, low SES.

**Table 5.** Weight of Spanish schoolchildren (kg), female series. Mean and sd, when available, extracted from each published article (references described in Table 1)

		Years of age										
Ref.		6	7	8	9	10	11	12	13	14	15	16
**1.**	m	19·0	21·5	23·6	26·1	30·4	31·2	35·7	38·3	42·1	–	–
	sd	1·9	2·3	3·0	2·8	6·4	4·9	5·9	5·3	6·0	–	–
**3.**	m	18·6	20·4	22·1	24·2	26·2	28·4	30·7	36·0	39·8	45·0	46·5
	sd	30	2·8	3·5	3·5	4·1	4·1	4·5	6·2	6·5	6·9	6·8
**4.a**	m	–	22·3	24·4	26·4	28·6	32·3	35·8	39·8	41·6	–	–
	sd	–	2·8	2·7	4·1	5·3	5·5	6·2	6·5	6·4	–	–
**7.**	m	18·1	21·7	24·2	27·1	29·8	33·2	36·7	41·3	45·0	–	–

**1.** Morros Sardá, 1934^(23)^, suburban, middle socioeconomic status (SES). **3.** Fernández Cabezas & Fernández Cabezas, 1946^(26)^, rural, middle SES. **4.a** Prevosti, 1949^(25)^, urban, low SES. **7.** Palacios & Vivanco (1965)^(29)^, rural, low SES.

As it has been noted above, weight data cannot be related to reference tables from WHO because the latter only include data below 10 years of age.

**Figure 5.**
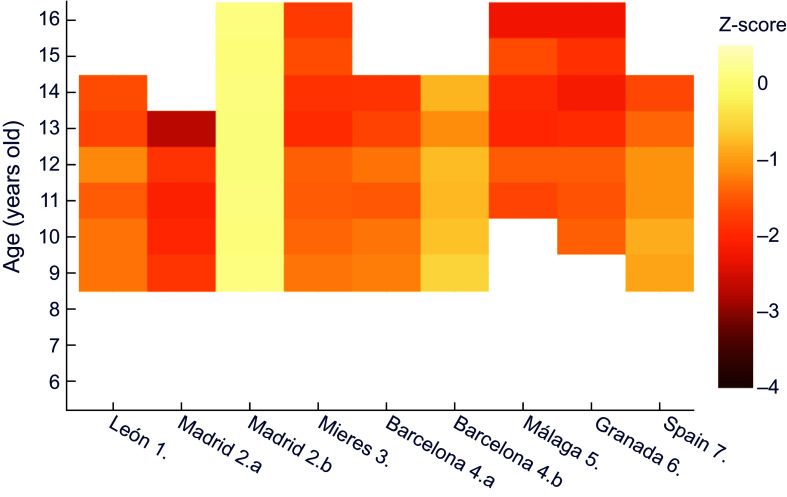
Z-score values for males’ weight for age, relative to the national reference^(32)^. **1.** Morros Sardá, 1934^(23)^, suburban, middle socioeconomic status (SES). **2.** Grande Covián et al., 1944^(24)^; (a) urban, low SES; (b) urban, mid-high SES. **3.** Fernández Cabezas & Fernández Cabezas, 1946^(26)^, rural, middle SES. **4.** Prevosti, 1949^(25)^; (a) urban, low SES; (b) urban, mid-high SES. **5.** Fernández Cabezas & Fernández Cabezas, 1952^(27)^, urban, mid-low SES. **6.** Fernández Cabezas & Fernández Cabezas, 1953^(28)^, urban, mid-low SES. **7.** Palacios & Vivanco (1965)^(29)^, rural, low SES.

A comparison of male weight for age in the historical series to contemporary Spanish data shows that the smallest discrepancies correspond to those of medium-high socioeconomic status in Madrid and Barcelona (Figure 5). Differences become more acute in all series after the age of 13, reaching in some cases values corresponding to severe underweight (z < –3). At the same time, it is again evident the influence of socioeconomic status in the datasets from subpopulations in Madrid^(24)^ and Barcelona^(25)^, as was observed in the height for age above.

**Figure 6.**
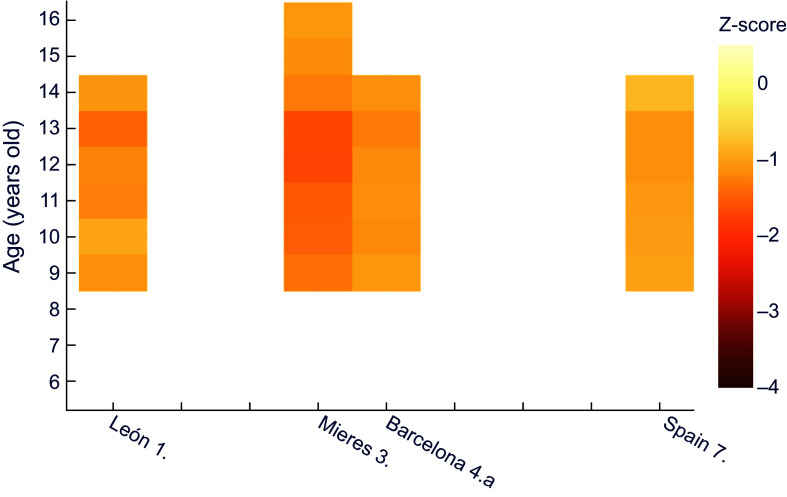
Z-score values for females’ weight for age, relative to the national reference^(32)^. **1.** Morros Sardá, 1934^(23)^, suburban, middle socioeconomic status (SES). **3.** Fernández Cabezas & Fernández Cabezas, 1946^(26)^, rural, middle SES. **4.a** Prevosti, 1949^(25)^, urban, low SES. **7.** Palacios & Vivanco (1965)^(29)^, rural, low SES.

With regard to females (Figure 6), the situation is notably better, since none of the series presents z-scores for weight below –1·5, the lowest values being found in Mieres and León between 11 and 13 years of age.

### BMI for age

**Figure 7.**
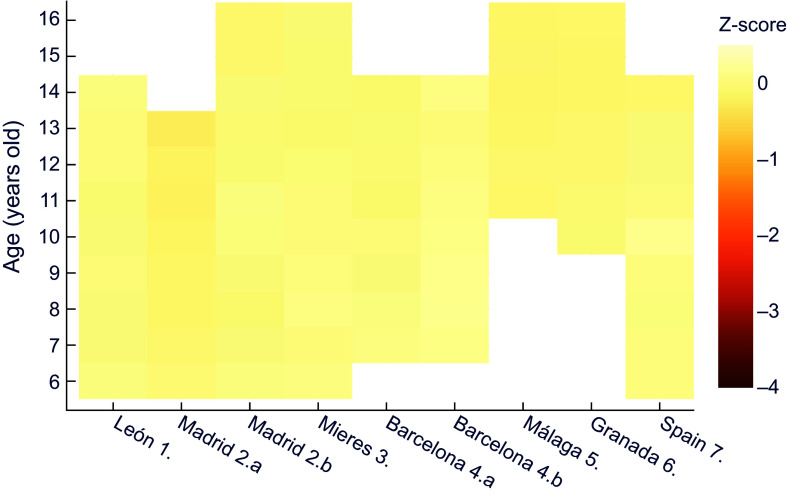
Z-score values for males’ BMI, relative to WHO^(30)^. **1.** Morros Sardá, 1934^(23)^, suburban, middle socioeconomic status (SES). **2.** Grande Covián et al., 1944^(24)^; (a) urban, low SES; (b) urban, mid-high SES. **3.** Fernández Cabezas & Fernández Cabezas, 1946^(26)^, rural, middle SES. **4.** Prevosti, 1949^(25)^; (a) urban, low SES; (b) urban, mid-high SES. **5.** Fernández Cabezas & Fernández Cabezas, 1952^(27)^, urban, mid-low SES. **6.** Fernández Cabezas & Fernández Cabezas, 1953^(28)^, urban, mid-low SES. **7.** Palacios & Vivanco (1965)^(29)^, rural, low SES.

**Figure 8.**
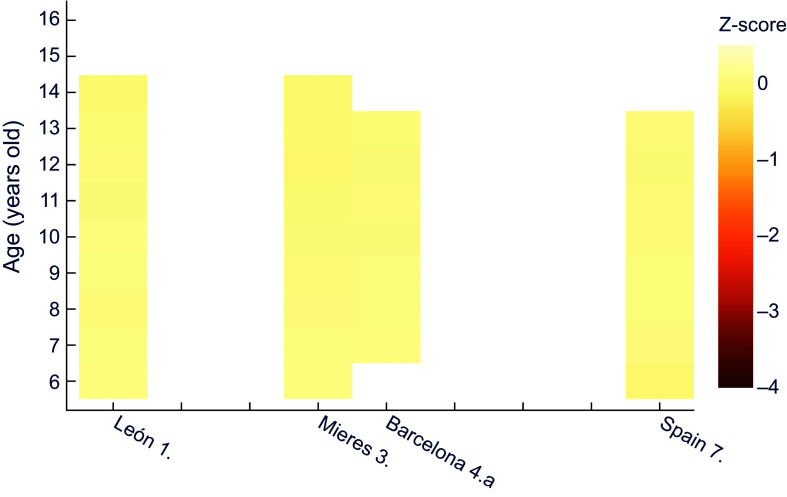
Z-score values for females’ BMI, relative to WHO^(30)^. **1.** Morros Sardá, 1934^(23)^, suburban, middle socioeconomic status (SES). **3.** Fernández Cabezas & Fernández Cabezas, 1946^(26)^, rural, middle SES. **4.a** Prevosti, 1949^(25)^, urban, low SES. **7.** Palacios & Vivanco (1965)^(29)^, rural, low SES.

**Figure 9.**
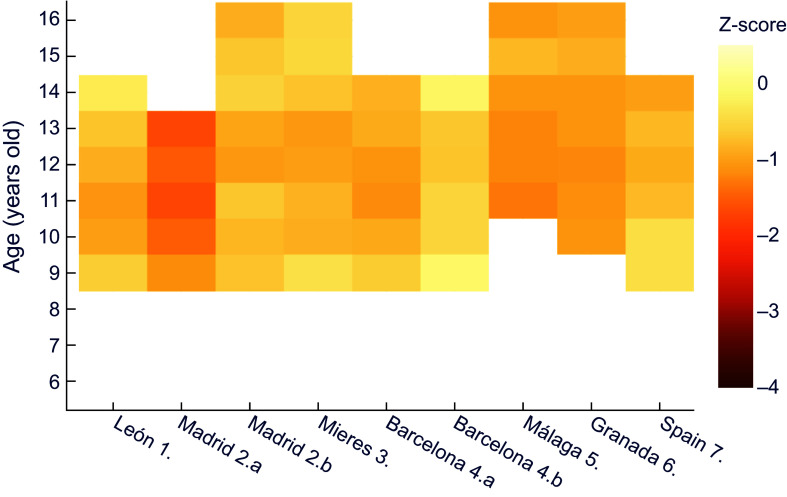
Z-score values for males’ BMI, relative to the national reference^(32)^. **1.** Morros Sardá, 1934^(23)^, suburban, middle socioeconomic status (SES). **2.** Grande Covián et al., 1944^(24)^; (a) urban, low SES; (b) urban, mid-high SES. **3.** Fernández Cabezas & Fernández Cabezas, 1946^(26)^, rural, middle SES. **4.** Prevosti, 1949^(25)^; (a) urban, low SES; (b) urban, mid-high SES. **5.** Fernández Cabezas & Fernández Cabezas, 1952^(27)^, urban, mid-low SES. **6.** Fernández Cabezas & Fernández Cabezas, 1953^(28)^, urban, mid-low SES. **7.** Palacios & Vivanco (1965)^(29)^, rural, low SES.

**Figure 10.**
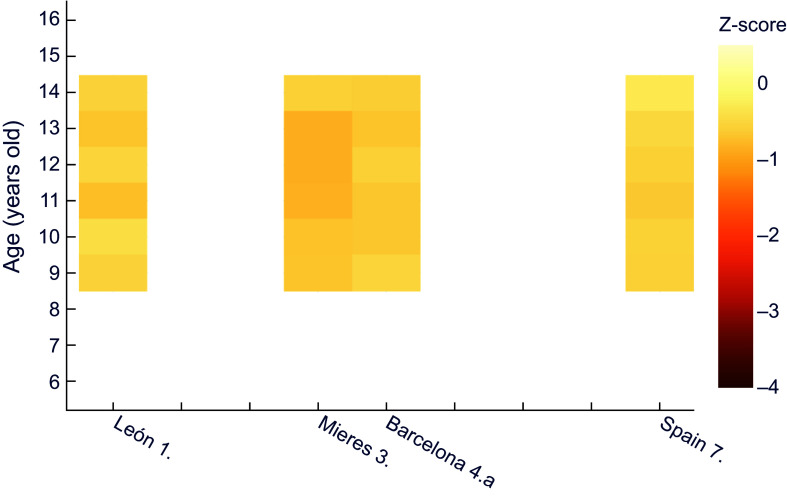
Z-score values for females’ BMI, relative to the national reference^(32)^. **1.** Morros Sardá, 1934^(23)^, suburban, middle socioeconomic status (SES). **3.** Fernández Cabezas & Fernández Cabezas, 1946^(26)^, rural, middle SES. **4.a** Prevosti, 1949^(25)^, urban, low SES. **7.** Palacios & Vivanco (1965)^(29)^, rural, low SES.

The BMI variable indicated no acute malnutrition in any male or female series, when contrasted with the international reference, with z-scores in all cases being close to zero (Figures 7 and 8). However, against the current Spanish reference some cases of wasting were ascertained in boys (Figure 9) but none in girls (Figure 10). Z values below −1 affected all male children in the Vallecas quarter, as well as those in Málaga (aged 11 to 14 years), Granada (aged 10 to 14 years) and in some cases at 12 or 13 years in León, Mieres, socially deprived Barcelona population and medium-high sector in Madrid. In general, the male series with the best socioeconomic situation in Barcelona was the one with the highest z-scores. BMI does not appear in the historical series, and here it has been estimated based on average values. For this reason, it is not being reported in tabular form.

## Discussion

Environmental conditions surrounding an individual, especially during the early stages of life, are key to the determination of body size, body composition and the correct function of the organism^(35)^. In addition, certain situations of poverty often converge with food insufficiency, lack of hygiene, low levels of education and even, on certain occasions, with child labour, all of which can lead to low height for the age or lead to either chronic malnutrition or retarded growth.

The results of the present study show, according to current references, a generalised stunting of schoolchildren during the period analysed; this situation, as is obvious, is especially marked in the most vulnerable groups from the socioeconomic point of view, that is, in those historical series which the authors present as poor, depressed areas. Similarly, in most of the historical series, a certain degree of underweight has been detected, which is more pronounced in areas of great poverty, such as the suburbs of the cities. However, BMI for age did not reflect acute malnutrition in any of the populations examined.

Even though the reports being compared are all transversal, the ages at which the highest z-scores were observed in both sexes approximately match the stage at which, in a general growth model, the pubertal growth spurt would take place, both longitudinally and in terms of weight. This may be due to the nutritional deficiencies experienced in early childhood, which resulted in a cumulative delay and were notably manifested at the time of greatest growth velocity, which coincides with the pubertal phase.

Likewise, the generalised presence of z-score values closer to the reference mean (in height, weight and BMI) in women supports the hypothesis of what has been called female eco-stability, which states that women are less susceptible to the influence of environmental factors during their ontogenetic development^(36)^. The problem is that anthropometric studies of girls are extremely scarce because in many cases, from the 1930s to the early 1960s, women had less access to educational institutions.

Thus, the Spanish education system developed by José Ibáñez Martín (first Minister of Education during the Franco regime) contributed to the social segregation of women in our country and hindered their access to higher education^(37)^. Consequently, as most of the anthropometric studies were carried out in these institutions, this makes hard to carry out an adequate analysis of the nutritional status of women in that epoch.

Indeed, the dictatorship maintained a hierarchical and paternalistic conception, prioritising values associated with men, so that men were constantly symbolised above women^(38)^. The subordination of women was inserted in an ideological conception consubstantial to Franco’s authoritarianism, which drank from the ideological sources of fascist virility^(39)^. The female role was relegated only to being a wife and mother, while all creative and intellectual capacities were reserved to the male intellect^(40)^.

Results in this study show that socioeconomically intermediate populations, corresponding to the suburban and rural areas of León and Mieres, present very similar z-score values (for both weight and height), despite their temporal difference of 12 years. This shows that, between 1934 and 1946, there does not seem to be a clear change in nutritional status, which means that there was no positive secular evolution for longitudinal and weight growth.

However, when comparing the z-scores of both variables among all series (except Madrid and Barcelona of medium-high status) and those corresponding to the whole Spanish territory, it can be seen that the values are quite disparate; this indicates that, from the 1960s onwards in Franco’s developmental period, a positive secular change in height and weight can already be glimpsed.

There were significant differences in the living standards of the different regions during the period analysed. Thus, the poorest areas were located in a strip stretching from the south-east to the north-west of Spain, while the richest areas were located in the north-east^(41)^. The considerable progress that Catalonia (in the north-east) underwent, due to its strong industrialisation, in comparison to other geographical locations, is evidenced by the results obtained in this study^(42)^. Indeed, although the populations of higher socioeconomic level in the large cities (Madrid, Barcelona) presented a very similar nutritional status, the chronic malnutrition and underweight of the most disadvantaged Catalan schoolchildren were much lower than that of the Madrilenians of the same social background.

This fact is reflected in the study by Palacios^(29)^ which, with a sample of 128 000 children from all over Spain, supports the aforementioned growth of Catalonia compared to other regions during the Franco regime. The more or less regular growth rate of schoolchildren in Barcelona observed in the forties was maintained until almost the eighties, despite the terrible living conditions experienced throughout the country^(43)^.

The situation of retarded growth and underweight in the Málaga and Granada series, although not as serious as in the case of Vallecas quarter in Madrid, seems to reflect the very poor socioeconomic situation in Andalusia. This means that, despite the fact that these populations have an average socioeconomic level, their nutritional status was on a par with or even slightly worse than that of the less favoured population of Barcelona. Similarly, certain studies reflect the low stature of Andalusian recruits due to the precarious economic situation of that region^(44)^.

On the other hand, results obtained in this work show that schoolchildren from rural areas such as Mieres had lower height and weight than those of schoolchildren from urban areas. This coincides with some investigations^(45)^ which show that, although the rural-urban gap was hardly significant during Franco’s autarchy, height was greater in urban areas than in rural ones. Although measurements of the scholars at this Asturian municipality are lower than those of Andalusian urban areas (Málaga, Granada) with similar economic development, this does not seem to be reflected in their nutritional status. Thus, the z-score values obtained are very similar in both rural and urban areas and some type of chronic malnutrition or slight or moderate underweight is perceived.

As reflected in the study of recruits from Madrid^(46)^, the results obtained in the present analysis showed the great heterogeneity and inequality in the population of large cities such as Madrid and Barcelona in the 1940s. The different living conditions between socioeconomic levels had a direct impact on the nutritional status of individuals, especially children. This fact has been evidenced in multiple studies at international level, like among Guatemalan children over four decades^(47)^, and also at national level^(24)^ whose authors pointed out that socioeconomic differences in the same territory could be even more important than hereditary factors.

The significant differences in height (10–20 cm) and weight (more than 10 kg) between contemporary Spanish schoolchildren and those of the different historical series, including those belonging to more favoured classes, are a reflection of the nutritional deficit in the population of our country. Nonetheless, they also show the evolution of the Spanish secular trend linked to the nutritional transition and the sociodemographic generational changes experienced since the 1960s. The arrival of immigrants, who began to gain weight especially from the 1990s onwards, also contributed to all of the above^(48)^.

These changes are also evident in the comparison of the average values for the historical publications analysed against the international references of the WHO^(30)^. However, it should be noted that this contrast tends to underestimate chronic undernutrition and underweight schoolchildren of both sexes, compared to a contemporary national reference^(32)^. The reason for this difference is that data from this global institution are elaborated with measurements of boys and girls, aged 5 to 19 years, combined from 3 original data sets^(31)^, collected in the 70s. However, the Spanish patterns used for comparison correspond to the general population of Madrid, which is more modern (with data collected from 2013 to 2015) and presents greater similarity from the genetic point of view.

A number of limitations may be considered for the current analysis. First, individual anthropometric data for the different historical series are not available, so comparisons had to be made based on the mean values, number of data points and sd of the variables analysed. Finally, it is important to note that, at the time when these historical studies were carried out, there was no anthropometric standardisation and, therefore, the measuring error among series could be considerably increased. Moreover, it would have been more appropriate to have larger samples, particularly of the female sex, and to have complete databases to improve the statistical analysis.

## Conclusions

A comparison of historical series published between 1944 and 1965 with the series measured during the 1930s, prior to the civil war (1936–1939), and with data collected more recently (2020) shows that the growth of Spanish children stagnated in the post-war period and that the secular evolution of height and weight occurred from the 1960s onwards.

The first decade post-war (data obtained between 1941 and 1946, published between 1944 and 1954) witnessed a prevalence of chronic undernutrition and underweight, categorised as mild or moderate, escalating to severe levels among the most economically disadvantaged social segments.

Longitudinal and weight growth were significantly more affected by socioeconomic conditions than by the level of urbanisation. Growth was especially impaired in the suburbs and among the poorer classes in large cities.
